# Topics and Insights on 1-D Tension Stiffening of an RC Member

**DOI:** 10.3390/ma19112303

**Published:** 2026-05-29

**Authors:** David Z. Yankelevsky, Yuri S. Karinski, Dina Tsemakh, Vladimir R. Feldgun

**Affiliations:** 1 Faculty of Civil & Environmental Engineering, National Building Research Institute, Technion-Israel Institute of Technology, Haifa 3200003, Israel; davidyri@technion.ac.il; 2 National Building Research Institute, Technion-Israel Institute of Technology, Haifa 3200003, Israel; tdina@technion.ac.il (D.T.); aefeldgo@technion.ac.il (V.R.F.)

**Keywords:** concrete, tension stiffening, uniaxial tension, bond–slip, cracks, stress redistribution, stochastic, deterministic

## Abstract

One-dimensional (1-D) tension stiffening is a fundamental behavior of structural concrete. It refers to the composite uniaxial behavior of a slender, symmetric concrete member of constant cross-section, bonded to a single reinforcing bar (rebar) along its axis. The rebar is subjected to tension by a pair of axial tensile forces applied at its ends. Despite the apparent simplicity of this configuration, the problem represents a cornerstone in RC mechanics. During the loading process, cracks are formed at different cross-sections along the structural member at stages where the tensile stress in the concrete at these cross-sections reaches the concrete tensile strength level. Each crack formation reduces the overall axial stiffness of the RC member, while inducing stress and strain redistributions in both the concrete and the rebar. The interaction between the concrete and the rebar is governed by the bond–slip relationship along their interface, which plays a critical role in controlling the transfer of stresses, the development of strains and the evolution of cracking. Most existing analytical and numerical models addressing this problem are based on simplifying assumptions assuming constant (deterministic) material properties and are denoted herein as “deterministic models”. Comparisons between analysis results of such models and experimental observations reveal substantial discrepancies in terms of the number of cracks, their spatial distribution, crack spacing, and the order of crack formation. Considering these inconsistencies, the present study postulates that the inherent variability of concrete properties, particularly its tensile strength, has a decisive influence on the structural response. To address this issue, the tensile strength of concrete is treated as a random variable characterized by the prescribed mean tensile strength and the coefficient of variation (CoV). The “stochastic analyses” with the variable tensile strength are conducted using an exact one-dimensional finite element formulation that explicitly accounts for discrete crack formation within the structural domain. These analyses yield results that differ markedly from those predicted by the deterministic approaches and exhibit characteristics that are in closer agreement with experimental evidence. These analyses indicate a more complex behavior of real structural members. It demonstrates that the CoV significantly influences the magnitude of cracking loads, crack locations, crack spacing, and the order of crack formation. The findings highlight the critical role of even slight material variability in tension stiffening behavior and justify the incorporation of concrete strength variability in tension stiffening modeling.

## 1. Introduction

### 1.1. General

**Figure 1:**
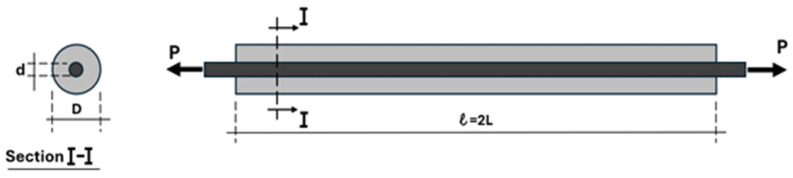
The tension-stiffening problem—scheme.

Consider a slender cylindrical reinforced concrete (RC) structural member of length l=2L and diameter D that is bonded to a concentric single steel rebar (diameter d) that is placed along the RC member axis of symmetry (Figure 1). The rebar is bonded to the surrounding concrete. The rebar ends are subjected to a pair of axial tensile forces P. The rebar is characterized by a high yield stress $f_{sy}$ and a comparatively small cross-sectional area $A_{s}$, whereas the surrounding concrete is characterized by a larger cross-sectional area $A_{c}$ and a significantly lower tensile strength $f_{t}$, defined herein as the mean tensile strength determined from experimental measurements.

The interaction between the rebar and the concrete cylinder is governed by the bond behavior at their common interface, typically represented by an experimentally determined bond stress–slip relationship. This relationship characterizes the dependence of the interfacial shear stress on the longitudinal relative displacement (slip) between the rebar and the surrounding concrete, thereby controlling the degree of composite action between these two sub-members.

In conventional analyses, concrete strength in compression and tension is considered constant, and homogeneity is assumed along the structural member. The composite action of the concrete–rebar structural member is calculated using the equilibrium and kinematic relationships, where the constitutive relationships for the rebar and the concrete in tension are assumed linear elastic until reaching yield in the rebar or cracking in the concrete. The analysis results in stress and strain variation along the longitudinal coordinate. The global equilibrium is also demonstrated by the sum of the axial forces carried by the steel rebar and the concrete at any cross-section, which remains equal to the applied load P. Consequently, the tensile force in the rebar at any given cross-section (e.g., I-I in Figure 1) is smaller than that in an isolated rebar subjected to the same axial load. This reduction in stress is accompanied by a corresponding reduction in elongation, such that the composite member exhibits a smaller axial deformation compared to the bare rebar. This enhanced stiffness is commonly referred to as tension stiffening. As the applied load increases, tensile stresses develop in the concrete, increasing from zero at the stress-free end sections to a maximum stress level at the mid-length of the member. At a critical load level, the tensile stress at the mid-section reaches the tensile strength of the concrete, leading to the formation of a transversal crack. This crack effectively divides the concrete member into two sub-members of equal length, which remain mechanically connected through the continuous rebar. New boundary conditions are formed at the newly opened crack faces, resulting in stress redistributions along the newly formed concrete sub-members, the rebar and the bond–slip along the interface. With a further increase in the applied load, additional cracks develop in a sequential manner. Each newly formed crack subdivides the existing concrete sub-members into shorter sub-members. This progressive cracking process continues until either the reinforcing bar reaches its yield stress, or the concrete sub-members become too short to sustain the development of tensile stresses sufficient to induce further cracking. During this process, the stiffness of the member gradually decreases, transitioning from the relatively high stiffness of the uncracked composite system to a state approaching that of the bare rebar at high loading levels, where the contribution of the cracked concrete becomes negligible [1,2].

Nevertheless, the concrete segments (sub-members) between cracks retain the ability to carry tensile stresses through their bond interaction with the reinforcement. This residual tensile resistance contributes to the overall stiffness of the structural member and governs both crack width development and total elongation under tensile loading. This behavior characterizes tension stiffening, which is a fundamental problem in reinforced concrete. It includes the major components of concrete and rebar stresses and their deformations, as well as the interfacial bond stresses and corresponding slips that govern the composite action. Yet it is a relatively simple problem since it refers to a uniaxial tensile stress problem.

The uniaxial tension stiffening is a fundamental mechanism that explains the concrete–rebar interaction under uniaxial tensile loading, assesses the cracking progress, the crack spacing and the crack width. These characteristics are the focus of the present investigation and are implemented in uniaxial concrete members as well as in 2-D panels subjected to in-plane loading, which are not included in the present investigation. Tension stiffening has also been incorporated into bending models, which are also beyond the scope of this study. The paper shows that the number of cracks, the crack locations, and the load levels at which cracks are formed depend on the specific distribution of the tensile strength along the member.

### 1.2. Modeling of the Tension Stiffening Problem

The general tension stiffening problem illustrated in Figure 1 is inherently two-dimensional, requiring the determination of displacement, strain, and stress fields as functions of both longitudinal and radial coordinates [3]. However, for slender members with relatively small width (or diameter), it is commonly acceptable to assume uniform stress and strain distributions across the cross-section. Under this assumption, the problem may be simplified to a 1-D formulation. Such 1-D models have been widely adopted in literature due to their analytical and computational simplicity and efficiency. In these models, both concrete and steel are typically assumed to exhibit linear elastic behavior up to their respective tensile strengths, characterized by Young’s moduli $E_{c}$ and $E_{s}$, respectively, and limited by the concrete tensile strength $f_{t}$ and the steel yield stress $f_{sy}$. The bond stress–slip relationship, however, introduces significant complexity into the problem. Experimental pull-out tests have shown that the maximum bond stress between steel and concrete depends on the compressive strength of the concrete and on the rebar confinement. The bond stress–slip relationship is highly nonlinear, exhibiting multiple stages including an initial nonlinear ascending branch, a plateau corresponding to maximum bond stress, a descending softening branch, and a residual frictional stage until the embedded rebar is completely pulled out. A representative monotonic bond stress–slip relationship has been described in the literature [4,5,6], capturing these stages. While such detailed models can be implemented in numerical simulations [7], most of the studies adopt simpler bond stress–slip models to allow simplified formulations and derive closed-form analytical solutions of the tension stiffening problem. Such models include a constant or piecewise constant relationship [8], a linear or a bi-linear [1] or a tri-linear [9], bond stress–slip relationship, and a nonlinear monotonic increasing bond stress–slip relationship [10].

To balance accuracy and allow analytical formulation, piecewise linear approximations of the full bond stress–slip relationship have been proposed [11,12]. These formulations allow the bond behavior to be represented by linear segments, enabling relatively simple analytical expressions while still capturing the essential features of the interfacial behavior. Most existing formulations of the tension stiffening problem [5,13,14,15,16,17,18,19,20,21] adopt a deterministic framework, assuming spatially uniform material properties along the structural member. Within this framework, one-dimensional modeling approaches have been extensively developed.

The deterministic formulation has been further implemented within an exact one-dimensional finite element framework. This unique approach is based on a multilinear representation of the bond–slip relationship and an exact analytical solution of the problem for each domain. The solution for each bond–slip domain refers to the entire member and allows the calculation of the stress fields, the cross-sections, and the displacements (elongations and slips) along its length. Formulating the problem to relate forces to displacements using a stiffness matrix allows for a configuration like a finite element formulation, although this involves a macro element and an exact solution, unlike assuming an approximate displacement field in a conventional finite element and the need to compact the element mesh to obtain a convergent solution. Formulating a comprehensive solution that incorporates all bond–slip regimes representing the entire bond–slip relationship allows accurate prediction of the behavior while using a limited number of elements, representing the different bond–slip regions along the member. This method can analyze both the pre-cracking stage [12,13] and the post-cracking response while loading up to the ultimate limit state [14].

The deterministic formulation allows precise solutions of general problems using a single finite element to represent the uncracked structural behavior having constant properties. When cracked concrete is considered, common numerical techniques prefer a smeared crack approach, which maintains the elements’ continuity and avoids increasing the number of elements and formation of new boundary conditions upon a new crack opening. The exact finite element formulation allows the development of a discrete crack opening at the exact location of the maximum tensile strength within the element domain, without the need for remeshing and adding more elements and degrees of freedom.

For that purpose, the stiffness matrix of any element contains the parameters of a potential discrete crack that opens within the element domain [14]. The exact finite element is subdivided into three parts: the narrow process zone at any coordinate where the tensile stress is about to reach the magnitude of the tensile strength stress and a crack is about to open, and two parts of the element at both sides of the crack. The local cracking zone of zero thickness may simulate a degrading stiffness or simply an abrupt transition to zero stiffness, where the tensile stress sharply drops to zero, and a full crack then opens. Formulation of the cracked finite element combines the two sub-elements connected to the longitudinal rebar through the bond–slip interface, where the continuous rebar interconnects the two sub-elements [14].

### 1.3. Characteristics of Deterministic 1-D Tension Stiffening

A typical slender RC member (Figure 1) with uniform tensile properties is considered, and basic characteristics of its response under increasing tensile loads applied at its ends are considered.

Tensile stresses in both concrete and rebar increase under increasing tensile load P. At low load levels (Stage 0, Table 1), the concrete remains uncracked.

At a critical load level $P_{cr1}$ (Stage 1), the first crack (crack 1) forms at the mid-length of the member, where the tensile stress reaches the concrete tensile strength. This crack divides the member into two identical sub-members of length L. With a further increase in load to $P_{cr2}$ (Stage 2), two additional cracks (crack 2) form at the midpoints of the existing sub-members, resulting in four equal segments of length L/2. At the next cracking stage $P_{cr3}$ (Stage 3), four additional cracks (crack 3) develop, subdividing the member into eight equal segments. This process continues until either the reinforcing bar yields or further cracking becomes impossible due to insufficient segment length.

At each stage, the crack spacing remains uniform, and an odd number of cracks is formed. The number of cracks $N_{C}$ is given by:

(1) $$N_{c}=\sum_{0}^{n}2^{n}-1$$

where n is the cracking stage number (column 1, Table 1).

Equation (1) calculates the development of one, three, and seven cracks during the first, second, and third cracking stages. Another important feature of deterministic models is that all crack widths are identical at a given load level. This is a direct consequence of the symmetry and uniformity of the problem, where each sub-member behaves identically. Therefore, crack width, which is the sum of slips at both sides of the crack, depends solely on the magnitude of the applied load and not on the crack spatial location [1,2,10,14].

These theoretically based models provide the distributions of the tensile stresses along the concrete structural member and the rebar, as well as the bond stress along the rebar-concrete interface at any load level.

**Figure 2:**
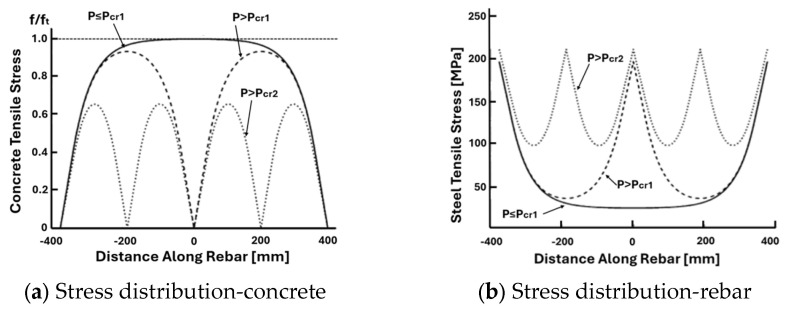
Stress distribution at several stages of loading.

In a specific tension stiffening problem that is described herein, a structural member with the following data has been analyzed: concrete cylinder length 2 L = 750 mm and diameter D = 90 mm, steel rebar diameter d = 12 mm. During the loading of this structural member, two cracking stages occur. Figure 2 depicts representative tensile stress distributions for several loading stages. Figure 2a shows the stress distribution in the concrete just prior to the first crack formation (solid line) and the redistributed stresses, right after the first crack formation (dashed line), where the tensile stress in the concrete at mid-span drops to zero (at the crack location). The stress distribution after the second stage cracking is also depicted (dotted line), indicating symmetrical identical distributions along the four sub-members.

Figure 2b depicts the corresponding stress distributions along the rebar. These distributions look like the inverse distributions shown in Figure 2a.

Multiplying the stress distributions in Figure 2a,b by the corresponding cross-section areas of the concrete member and the steel rebar, respectively, will yield the axial force distributions in both components. The inverse shapes mean that the summation of the corresponding force curves in both figures will yield a constant force that is equal to the applied load P.

Although equilibrium indicates a constant axial force at any cross-section along the structural member, the stiffness of the member decreases with increasing the number of cracks.

**Figure 3:**
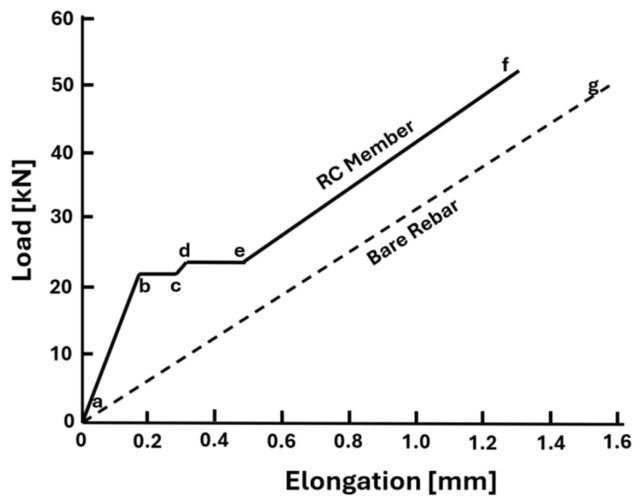
Load–elongation relationship.

The above cracking process is complemented by the load–elongation relationship. Figure 3 depicts a scheme of the force–elongation (the member extension) relationship: The solid staircase shape line describes the member response, and the dashed line presents the load–elongation relationship of the bare bar. A pronounced higher stiffness (a–b) of the composite member at the uncracked state is observed, compared to that of the rebar (a–g). A single crack opens at “b” (corresponding to stage 1-Table 1), and b–c represents the crack width, which yields a jump in the member elongation. Further loading along c–d is characterized by reduced stiffness and continues until two more cracks open in the following cracking stage (d–e), corresponding to stage 3; Table 1. The following loading of a member with three cracks (e–f) in the present numerical example is characterized by an even lower stiffness, that is, close to the stiffness a–g of the bare rebar, and is considerably smaller than the initial stiffness of the composite member a–b.

### 1.4. Experimental Investigations

Experimental investigations have been reported in the literature with different levels of detailing. In theory, much information could be gathered during a tension-stiffening laboratory test (e.g., strains along the concrete and the rebar, crack locations, crack width variation, overall elongation), but in practice most experimental reports depict the cracking pattern, e.g., [22,23,24], and the monitoring system measures the overall force–elongation relationship. The overall elongation, which is simple to monitor, implicitly includes the sum of crack openings. A common testing program does not follow the continuous opening and growth of every single crack, mainly because the exact crack location is unknown a priori. Otherwise, small size sensitive LVDT transducers could be attached to both sides of an expected crack location. In several test programs, dial gauges were used at discrete steps of the loading process to measure the average strain within a relatively coarse grid of reference markers on the specimen surface [25]. The major disadvantages of such discrete measurements are the limited accuracy, the inability to isolate the crack extension only, and the lack of a continuous record that is essential for a precise follow-up of the crack width.

In that respect, advanced theoretical models may significantly enhance the insight gained on the tension stiffening problem, as they may continuously follow all the above variables and clarify major characteristics of the member response. Thus, it may help focus on new experimental strategies to measure these features during an experimental program. Such synergetic research progress on the problem may considerably advance our understanding of the problem, provide new experimental data, and help to further improve the theoretical models.

A few test reports provided photos of the cracked members, mostly depicting the final state of cracking but sometimes also marking the crack number, indicating its order of appearance. These photos indicate that crack spacing is not even, and the order of crack appearance is unexpected and entirely different from the expected cracking pattern characteristics obtained from the deterministic models (Table 1). The total number of cracks that are obtained in similar tested members may be different and may be either even or odd numbers, different from Equation (1).

**Figure 4:**
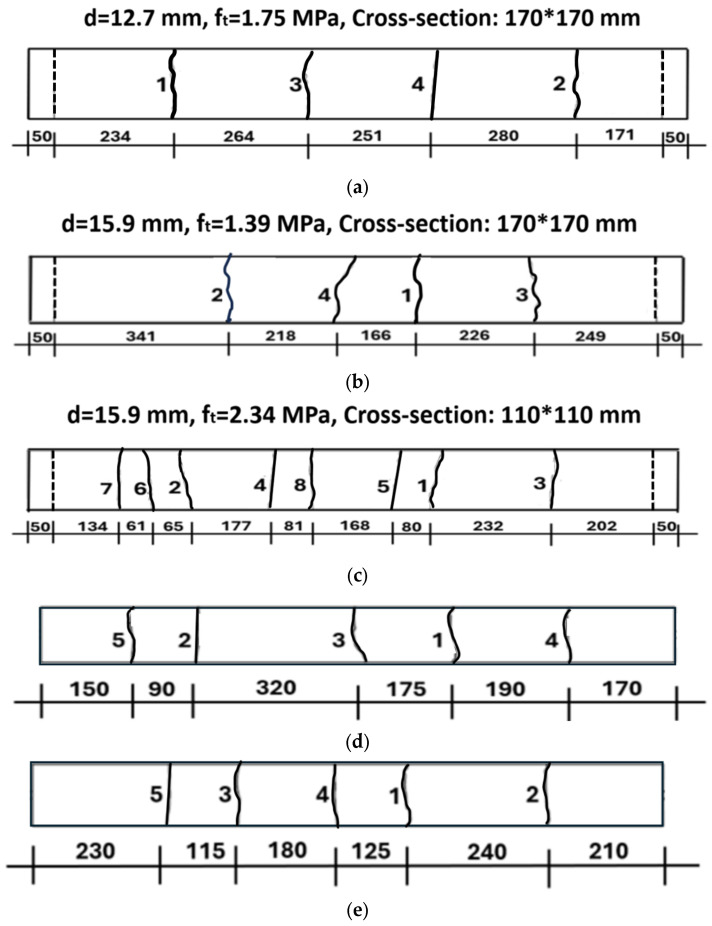
Tested specimens cracking (spacing in [mm]). (**a**) Specimen 13-170 [26]. (**b**) Specimen 16-170 [26]. (**c**) Specimen 16-110 [26]. (**d**) Specimen STN12 [27]. (**e**) Specimen STN16 [27].

Munoz [26] conducted tests on concrete specimens with a length of 130 cm and square cross-sections, each reinforced with a centrally embedded FRP bar. The results (Figure 4a,b) show that two specimens with different reinforcement diameters and tensile strengths both developed four cracks (an even number), yet exhibited markedly different crack locations, spacing, and orders of formation. In another specimen with a smaller cross-section (Figure 4c), eight cracks were observed.

Similarly, Wu [27] investigated specimens 110 cm in length with square cross-sections (100 mm × 100 mm), with identical tensile strength values ($f_{t}=2.04$ MPa). The specimens were reinforced with steel rebars of different diameters, 12 mm (Figure 4d) and 16 mm (Figure 4e). Five cracks are depicted in each specimen, but with entirely different orders of crack formation. Furthermore, measurements of crack widths revealed significant variability.

These examples clearly prove that the number of cracks cannot be determined a priori and that the order of their appearance is random.

These experimental findings clearly demonstrate that crack spacing is non-uniform, crack widths vary significantly, the order of crack formation is unpredictable, and the total number of cracks cannot be determined a priori. It turns out that tests on similar specimens yield different results. Consequently, commonly used concepts such as “average crack spacing” and “average crack width,” which are prevalent in design codes and deterministic analytical models, are not fully consistent with observed physical behavior.

These discrepancies between deterministic predictions and experimental observations strongly motivate the introduction of stochastic aspects to capture the inherent variability of the system.

## 2. Stochastic Approach

### 2.1. General

Deterministic approaches are widely used in structural engineering analysis due to their simplicity and computational efficiency. In such approaches, material properties are represented by single characteristic values, often corresponding to mean or characteristic strengths.

In reliability-based design, probabilistic considerations are sometimes introduced using fractile values (e.g., 5% fractile strength is a characteristic strength below which only 5% of all potential strength results are expected to fall). However, these values are typically incorporated into otherwise deterministic analyses and do not fully capture the spatial variability of material properties. A more rigorous stochastic approach considers material properties as random variables or random fields, governed by specified probability distributions. In the context of concrete, both normal [5,28,29,30] and log-normal [28,31,32] distributions have been used to describe variability in material properties. Stochastic modeling may be applied at different scales:

- **Macro-scale models**, where variability is introduced at the structural level, allow efficient simulation of phenomena such as multiple cracking, crack distribution, and crack sequencing [33,34]. Such approaches have been successfully applied to tension stiffening problems involving fiber-reinforced polymers (FRPs), fiber-reinforced concrete (FRC), and strain-hardening cementitious composites [31,35], as well as to crack localization problems [36,37].
- **Meso-scale models**, considering aggregates and interfaces, and **Micro-scale models**, capturing detailed material heterogeneity [29,38,39], may refer to the aggregate size, interfaces, etc.

Stochastic analysis has also been applied to FRP and FRC tension stiffening problems [31,35] and tensile ductility [38], including the cracking localization problem [36,37].

The present study adopts a macro-scale stochastic approach, focusing on the variability of concrete tensile strength at the cross-sectional level [33,34]. While stochastic modeling of bond–slip behavior has also been investigated [30,40,41,42,43,44], it should be mentioned that the magnitude of the bond strength depends on the compressive strength of concrete and on the concrete cover of the pulled-out rebar. Therefore, its inclusion as a stochastic variable is irrelevant. Furthermore, simultaneous variation in multiple parameters may obscure the individual effects of each parameter. Accordingly, the present investigation isolates the tensile strength as the sole stochastic parameter, allowing a clear assessment of its influence on the structural response.

Within this framework, each cross-section of the structural member is assigned a distinct tensile strength value. In the finite element discretization, this corresponds to assigning a unique tensile strength to each element. This results in a refined mesh of elements, even though that approach provides exact solutions for a deterministic problem using a small number of elements, as explained above. That aspect will be further discussed in Section 3.1. In the following investigation, the extended exact one-dimensional finite element formulation that had been used for deterministic analyses [14], will be extended for accounting for spatially varying tensile strength distributions, by using a finer mesh of elements, where each element will be assigned with a different tensile strength, that is derived from the stochastic tensile strength distribution, based on the given mean tensile strength and assuming a normal distribution of the tensile strength with a pre-determined coefficient of variation (CoV). This extended model will be denoted “the stochastic model”.

Experimental evidence indicates that the coefficient of variation (CoV) of concrete tensile strength typically ranges between 10% and 15% [28,31,45], with some studies reporting even higher values [3,33,46]. Based on these findings, the present study considers CoV values up to 20%.

It is noted that while long-term effects (e.g., creep) may reduce the mean tensile strength, the CoV remains approximately unchanged [47].

Although the tensile strength of concrete is often neglected in strength-based design due to its relatively low magnitude compared to compressive strength, it plays a critical role in performance issues (except for containers, tanks, etc.), and the major concern is that the crack width should satisfy a certain standard for the allowable width criterion. Crack width formation and growth are one of the products of a tension stiffening analysis; it is presented in Section 3.6.

In deterministic analyses, the constant magnitude of tensile strength affects the cracking load but does not influence the cracking pattern. In contrast, in stochastic analyses, spatial variability in tensile strength, even if limited variation is considered, has a profound impact on the entire cracking process and consequently the tension stiffening behavior, including the crack initiation load and location, and the following order of cracking. This behavior likely represents more realistic behavior, such as that observed in a series of tests on concrete members.

To focus on the tensile strength variation along the structural member and assess its effects on the member response, and to keep a relatively simple and clear methodology, we assume that the other concrete-related parameters (i.e., Young’s Modulus *E_c_* and the bond stress–slip relationship) remain constant. It is important to note that isolating a single parameter from all others and examining its stochastic behavior effect only is a unique advantage of a theoretical model that cannot be examined in any realistic test, not to mention the major problem of experimentally determining the magnitude of the local variable parameters.

### 2.2. Problem Modeling

Several major aspects of problem modeling are outlined in the following.

#### 2.2.1. The Bond Stress–Slip Relationship

**Figure 5:**
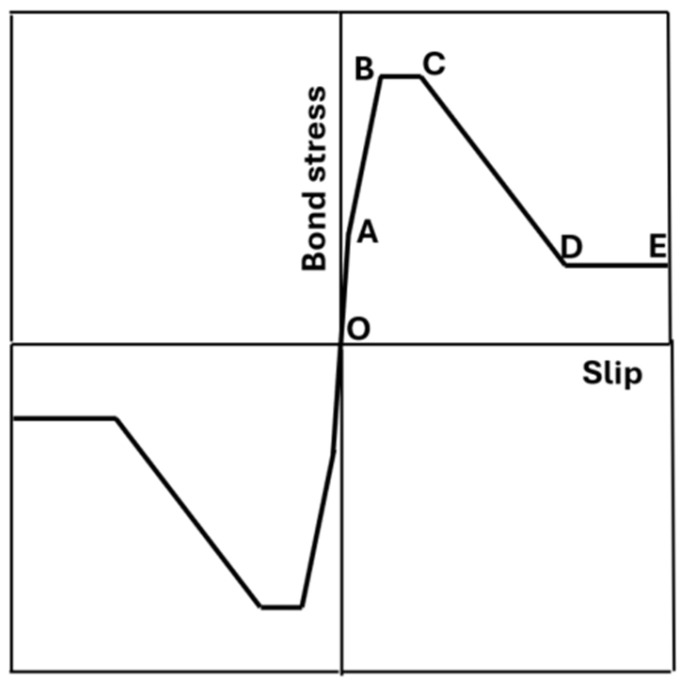
The bond loading path of the stress–slip relationship.

To closely represent the complete monotonic bond stress–slip relationship, a piecewise linear model is suggested (Figure 5) to closely represent the experimental behavior of pull-out tests [9,11,31].

It is composed of a bi-linear relationship (O–A–B) ideally describing the ascending nonlinear branch, followed by a constant segment (B–C) that precedes a descending branch with a negative stiffness (C–D) that is connected to a constant residual friction segment (D–E).

#### 2.2.2. The Variation in the Tensile Strength

The probabilistic nature of the tensile strength of concrete induces a significant strength scatter along the structural member length [33,35,48,49]. The strength variation is described by a normal distribution [3,20,30] with an average strength *f_t_* and a coefficient of variation CoV. Based on Section 2.1, this study aims to consider normal distributions of the tensile strength for three values of the coefficient of variation (CoV): 5%, 10%, and 20%, covering the common range of tensile strength variation. Refer to a given tension stiffening problem with a given average tensile strength *ft*. A given structural member is discretized into N finite elements; each element is assigned a randomly generated tensile strength value consistent with the prescribed statistical distribution. Random values are generated using the Ziggurat algorithm implemented in MATLAB R2022b [50].

The larger the number of elements, the finer the mesh and the more detailed the strength distribution. The macro-scale analysis used in the present study, as well as in numerous other structural engineering studies, aims at consideration of structural member performance, using a homogeneous continuum representation of the medium and assigning engineering physical parameters to represent strength and deformation. Therefore, in the geometrical modeling of the stochastic problem (Section 3), it has been determined that the mesh size should not be smaller than several mm, that are 1–2 times the aggregate size scale.

## 3. Stochastic Analysis

### 3.1. General

In the following analysis, a stochastic analysis is carried out for the same problem presented in Section 1.3, for which a deterministic analysis has been carried out (Figure 2). The same major parameters of this member are used: length 2 L = 750 mm, diameter D = 90 mm, and the centrally embedded steel rebar diameter d = 12 mm. The Young moduli of concrete and steel are 29 GPa and 210 GPa, respectively; the concrete strengths in compression and tension are 33 and 3.1 MPa, respectively. The yield stress of the steel rebar is 460 MPa. The bond stress–slip coordinates (t, s) in Figure 5 are described by the characteristic points depicted in the figure: A (6 MPa, 0.03 mm), B (12 MPa, 0.6 mm), C (12 MPa, 1.0 mm), D (5 MPa, 3.0 mm).

The analysis of a problem followed an incremental loading protocol until reaching the rebar yield stress at the steel rebar ends (P = 52 kN). The stochastic analyses herein refer to a mean tensile strength of *f_t_* = 3.1 MPa, where its stochastic variation along the structural member is examined for three different levels of CoV: 5%, 10%, and 20%.

The structural member has been discretized into N = 160 finite elements to allow a fine tensile strength variation along the member. To examine aspects of stochastic behavior, 30 analyses have been carried out (i.e., 10 different structural members were analyzed for each of the 3 CoVs). The analysis results of the 30 calculations (cracking loads and members’ elongation) are presented in the following.

### 3.2. Number of Cracks and Their Locations

**Figure 6:**
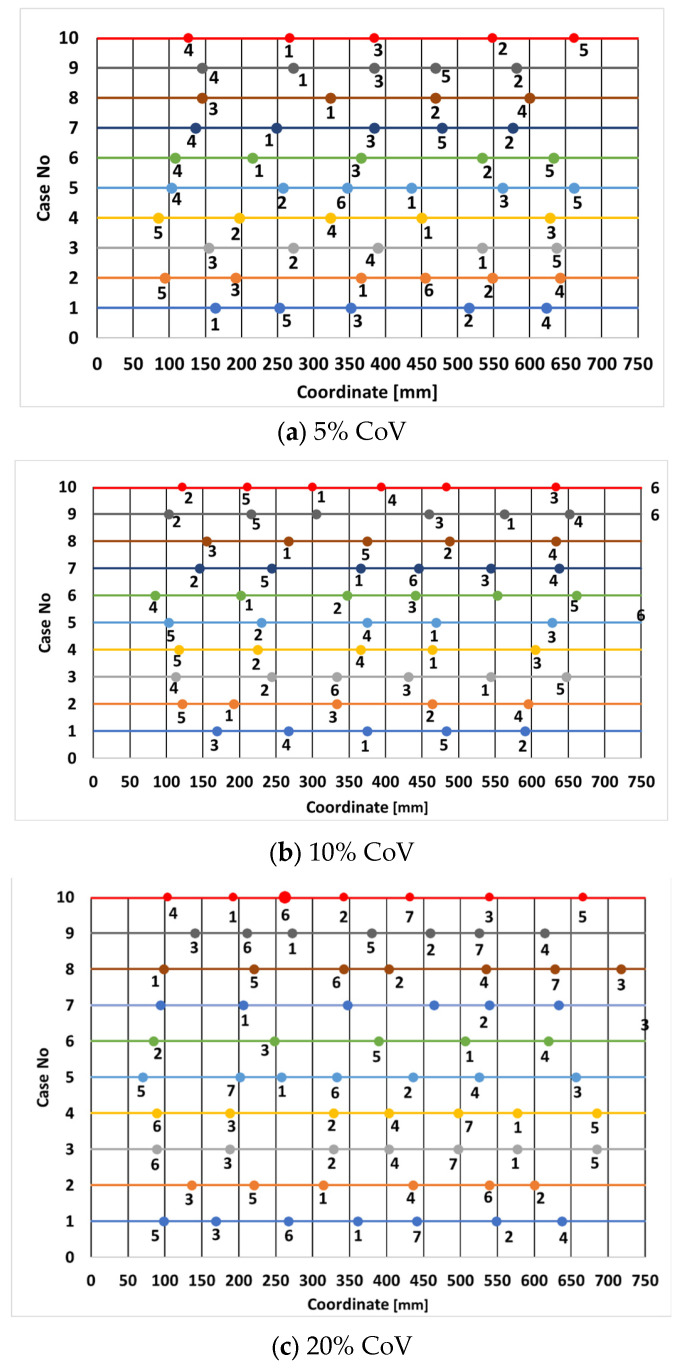
Cracks’ locations and order of their appearance.

Figure 6 depicts the results of the 30 analyses. Ten colored lines are depicted for each CoV, representing the 10 apparently identical structural members, each with a different stochastic distribution of concrete tensile strengths. Each colored dot along a line (a member) indicates the coordinate at which a crack was formed, and the number next to that dot denotes the order of appearance of this crack during the loading process. The analysis shows that 3–7 cracks open in a particular case for both examples.

Table 2 presents the number of cases where a certain number of cracks (NC) are formed for each CoV.

Figure 6 and Table 2 clearly show features of stochastic analysis that are considerably different from the deterministic analysis:

- Even a slight variation in the tensile strength along the member (CoV = 5%) yields an entirely different behavior compared to that obtained in the deterministic analysis.
- A larger number of cracks are developed for a higher CoV, i.e., for a higher degree of variability of the tensile strength along the structural member.
- Both odd and even numbers of cracks may open for the same CoV.
- The order of crack development cannot be predicted a priori.
- The distance between adjacent cracks is not equal.

These early results already prove that the CoV of the tensile strength has a major effect on the structural member response.

The following sections aim at gaining more insight into the CoV effects on the structural member cracking.

### 3.3. The Cracking Loads

Table 3, Table 4 and Table 5 present the cracking load for every crack in the 10 different cases of each of the three CoVs. The columns refer to the crack number according to the order of appearance, and the cracking loads increase with an increase in the crack number. Each column presents the cracking load corresponding to another crack in the order of its appearance. Opposed to the deterministic case (denoted “Det” in Table 3), every crack appears at a different load level. The minimum, maximum and average cracking loads for this crack number are specified. The data is compared with the deterministic solution results of the same problem, where a constant average tensile strength of the same magnitude as *f_t_* that is the mean tensile strength in the stochastic case, is assumed along the member.

**Figure 7:**
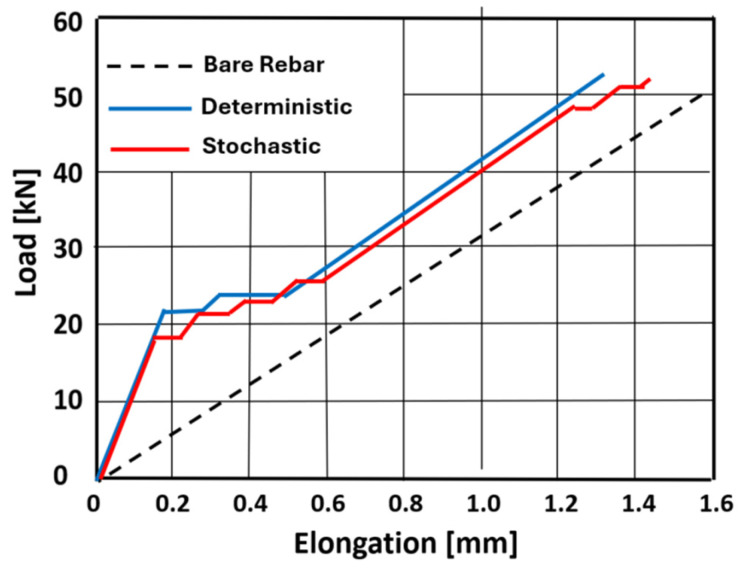
Deterministic and stochastic load–elongation relationships (Case 5, CoV = 5%).

Table 3, Table 4 and Table 5 provide the following insights:

- For the same average tensile strength, more cracks are developed in the stochastic case compared to the deterministic case.
- More cracks are developed where the CoV is larger.
- The cracking loads in the stochastic cases are considerably lower than those in the deterministic case. The larger the CoV is, the lower the magnitudes of the cracking loads are.
- Compared to the narrow range of cracking loads in the deterministic case, the range of cracking loads in the stochastic case is considerably larger.
- The ratio between the average cracking load and the deterministic cracking load (last row in Table 3, Table 4 and Table 5 decreases with the CoV for a given crack number: for example, for the 1st crack, this ratio is 88%, 75.3%, and 52.9% for CoV = 5%, 10% and 20%, respectively. This indicates that larger CoVs are associated with smaller cracking loads.
- The larger the number of cracks and the wider the range of cracking loads, the more refined the staircase-shaped force–elongation curve in the stochastic case. Figure 7 depicts the force–elongation relationship for Case 5 (CoV = 5%) for the stochastic analysis, in comparison with the result for the deterministic analysis. It clearly shows that the first crack in the stochastic case occurs at a lower load level compared to the deterministic case. The stochastic analysis yields a multi-stair curve, which is mostly below the deterministic curve, and the fifth and sixth cracks open at very high loads.

### 3.4. The Critical Cracking Tensile Stresses

**Figure 8:**
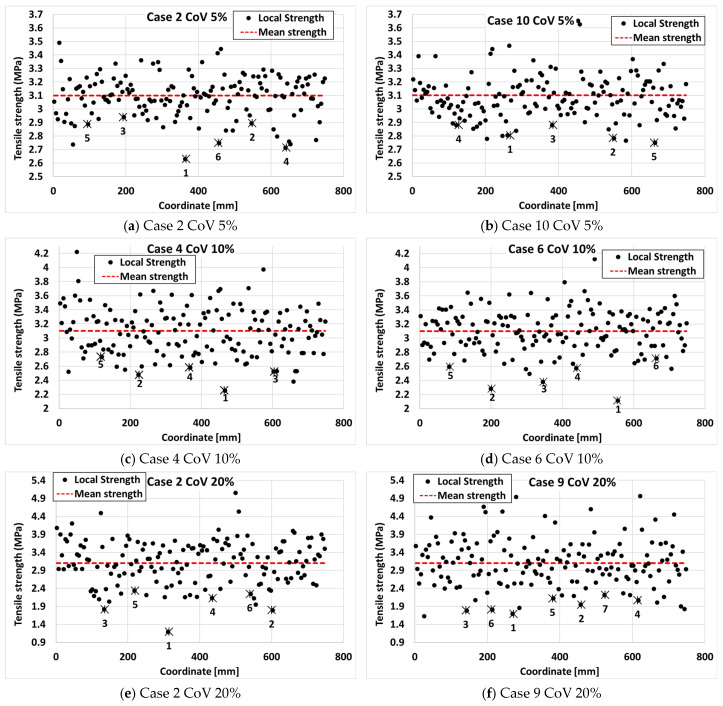
Stochastic tensile strengths and cracks’ locations.

This section provides more insights into the CoV effect on the smaller cracking loads in the stochastic case. Two arbitrarily selected cases are presented for each of the three examined CoVs. In each of the six cases shown in Figure 8a–c, the stochastic tensile strengths in all finite elements are depicted in black dots. The expected larger scatter of the stochastic tensile strengths for larger CoVs is clearly observed. For comparison, the mean tensile strength (*f_t_* = 3.1 MPa) is depicted in a dashed red line.

Analysis of these cases yields the location of cracks, which are depicted with the ∗ sign, indicating both the crack location and the magnitude of the local tensile cracking strength. The number next to the ∗ sign indicates the crack number, related to the order of crack formation.

It is interesting to note that all the critical cross-sections at which cracks open are associated with tensile strengths that are at the lower bound of the stochastic scatter. The larger the scatter (larger CoV), the lower the magnitudes of the cracking stresses. These interesting findings are in accordance with the relatively lower cracking loads in the stochastic analyses that are presented in Table 3, Table 4 and Table 5.

It is important to note that although the stochastic tensile strengths have been produced from a symmetrical normal distribution, it turns out that only the lower strengths affect the tension stiffening behavior.

It is also important to note that these cracked cross-sections cannot be determined a priori, because each cracking stage produces a new redistributed state of stress, which affects the location of the following cracked cross-section. That means that, as opposed to the deterministic analysis where all crack locations could be determined a priori, in the stochastic case, the history of cracking development determines the following crack appearance.

### 3.5. Strain and Slip Distributions—Stochastic and Deterministic Analyses

**Figure 9:**
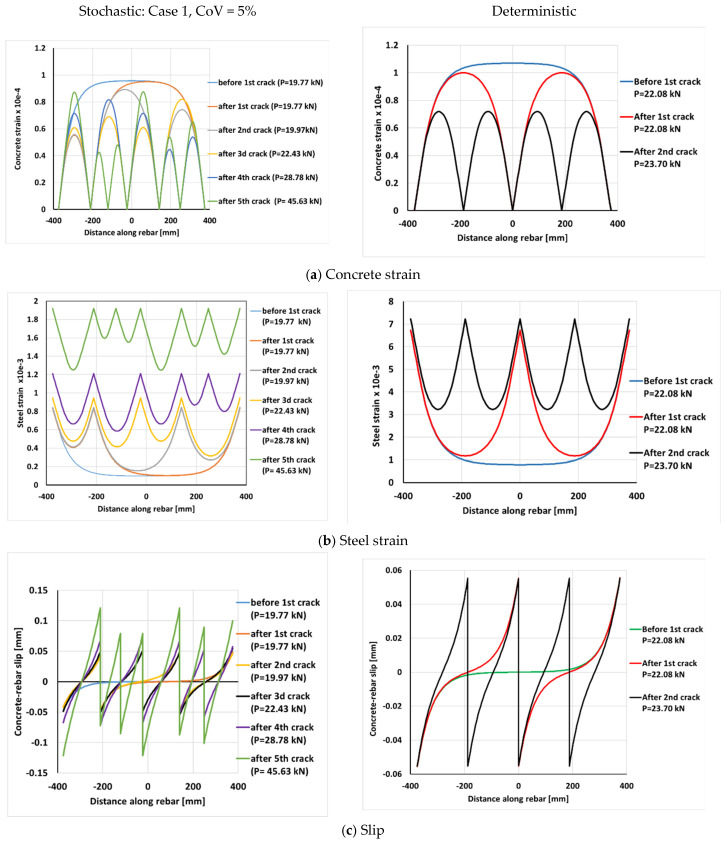
Example of tensile strains and slip distributions.

This section presents typical tensile strain variations along the concrete cylinder and the steel rebar, and bond–slip variations along the concrete–rebar interface, for representative cases. Figure 9 shows representative results for Case 1, CoV-5%. It should be noted that the stochastic model solution procedure checks the likelihood of a new crack development within each element at each loading increment throughout the analysis.

To appreciate the strong effect of the tensile strength variation on these results, even in the case of relatively small CoV (5%), these results are compared with the results of the deterministic case, assuming a constant tensile strength with the magnitude *f_t_* that is the average strength in the stochastic analysis.

Figure 9a depicts the concrete strains in the stochastic and deterministic cases at several stages of loading, related to different crack openings. Similarly, Figure 9b describes the local strain distributions along the rebar, and Figure 9c describes the local slip along the concrete–rebar interface.

Compared to the deterministic strain distributions in the concrete member and the steel rebar, the stochastic strain distributions (Figure 9a,b) are more complex and non-symmetrical with respect to the structural member mid cross-section. This illustrates the complexity of the stochastic results compared to the deterministic, straightforward solution.

### 3.6. Crack Width Formation and Growth

During loading of the structural member, cracks are formed at different load levels, and each cracking stage is associated with redistribution of the axial stresses and strains, as well as the bond stresses and the corresponding slips, as discussed and demonstrated in Section 3.5.

The deterministic solution of the slip distribution at several loading stages (right-hand side—Figure 9c) shows equal slips on both sides of each crack. This is an expected result due to the identical length of all sub-members (two or four) that are formed upon formation of a single central crack (at P_1_ = 22.08 kN) and after formation of two more cracks (at a slightly higher load P_2_ = 23.7 kN), thus splitting the member into four identical sub-members. The symmetrical sub-members at each load level lead to identical slips at the cracks’ boundaries. It may be noted that after the second cracking level, the slip at each crack boundary is slightly larger than the slip at the first cracking level, due to the slightly higher load (P_2_ compared to P_1_), but this slight difference is not noticeable on the figure scale. Since crack width is equal to the sum of slips on both sides of a crack, it follows that just after formation of the four cracks (dotted line—right-hand side of Figure 9c), the crack widths are equal.

In contrast, the stochastic solution depicted on the left-hand side—Figure 9c—is more complex. Different slips are developed on the sides of each crack due to the different sub-member lengths, which change upon each following crack formation. This leads to complex crack-width variations that cannot be assessed beforehand but can be fully followed using the present approach.

## 4. Conclusions

Tension stiffening is a well-known problem in structural concrete, which represents the coupled behavior of a steel rebar embedded in a slender concrete member under axial tensile loading. Common solutions to the problem are based on the mean tensile strength of concrete (deterministic approach) and yield simple and clear results. However, comparison of the deterministic model features with test results shows major differences in terms of the number of cracks, the order of cracking, spacing between cracks, etc. To bridge these differences, a more realistic model is required.

The present study provides a comprehensive stochastic investigation of tension stiffening in reinforced concrete members, extending classical deterministic formulations by incorporating spatial variability in tensile strength. This stochastic approach implements a previously developed exact finite element formulation of deterministic tension stiffening, including possible development of a discrete crack within the element domain. The stochastic approach accounts for the local tensile strength variation, which is assigned to each element, based on the normal distribution of tensile strength with a given average tensile strength and assuming three levels of the coefficient of variation (CoV). Analysis results yield similar features to those observed in experimental investigations. Three levels of CoV were examined, and ten cases were analyzed for each CoV. The analyses yield the stress, strain and displacement fields in the concrete and along the rebar, the interfacial bond stress and slip displacement along the concrete–rebar interface and calculate the overall force–elongation relationship.

The fundamental Behavior has been clarified. It is shown that tension stiffening is governed by complex interactions between cracking, bond–slip, and stress redistribution, and that attempts to use simplified deterministic models significantly oversimplify this behavior.

A comprehensive investigation considering the stochastic variation in the tensile strength along the structural member of a typical problem, although more complex, provides realistic results that are in accordance with experimental findings and yields new insights, such as:

- Similar tests may yield different crack patterns and a different number of cracks for a given CoV.
- The number of cracks may be either even or odd, as observed in different experimental investigations, and differently from the odd number only, which is obtained in deterministic modeling.
- A single crack is commonly developed at a location that cannot be a priori expected along the concrete member, opposed to a crack that is opened at an a priori determined cross-section in the deterministic case, and a number of cracks that open simultaneously at the centers of the sub-members, in the deterministic analysis, compared to single crack which opens in turn at a different load level during the continuous load increase in the stochastic case.
- The order of crack formation cannot be predicted beforehand.
- The cracks are not evenly spaced, and their widths are different.

These observations were obtained from modest samples of 10 specimens examined for each CoV. This size is sufficient for demonstrating major new aspects of stochastic behavior, such as the effect of CoV on the cracking stresses, the sequence of cracking, and crack spacing. Larger samples would not yield more fundamental characteristics of the described behavior.

The present investigation has demonstrated the major effect of the variable distribution of the tensile strength of concrete along the structural member. For identical average tensile strengths, larger CoVs yield lower magnitude cracking stresses at the cracked cross-sections. It means that the CoV is more important than the average tensile strength; a lower average tensile strength with a smaller CoV may yield a better performance than a higher average tensile strength associated with a larger CoV. Therefore, attention should be given to improved concrete mixtures and concrete production, placement and curing, to ensure lower CoVs, thus exploiting the concrete significantly with the specified average strength and enhancing the structural performance.

It should be clarified that the tension stiffening problem addresses the performance aspects of the 1-D member, through the cracking development, the stiffness decreases and the related elongation variation with loading. The capacity of the analyzed member is beyond the scope of our considerations, as it solely depends on the rebar yield stress, disregarding the composite action and the role of concrete.

Several of the engineering implications of the present study are:

- The concept of “average crack spacing” is not physically representative.
- Deterministic models may overestimate cracking loads.
- Structural performance depends more on variability control than on mean strength.

Although not aiming to address a practical problem, but rather to enhance understanding and gain new insights on the uniaxial tension stiffening behavior, important practical recommendations may be drawn. Improved quality control of concrete may help to predefine the concrete quality and affect the CoV; control to reduce CoV may yield higher effective cracking resistance, more predictable structural behavior and improved serviceability performance.

This study also clarifies different difficulties that emerge. For example, it is extremely difficult or even practically impossible to validate the theoretical model results through a simple test program, for several reasons: the need of a sufficiently large number of similar specimens to allow the probabilistic analysis of the experimental results; the major difficulty to a priori identify the tested specimen stochastic properties through nondestructive tests, such that these properties are properly included in a stochastic model, etc. Simpler challenges that may be addressed rather easily include improved monitoring systems in experimental programs to provide quality data that can be compared with advanced models. The complex behavior of cracking development requires more attention for better, more realistic determination of design parameters to replace commonly used terms like “the average crack width” and “crack spacing”. These thoughts and others will be considered in the following stages of this investigation.

## Figures and Tables

**Table 1:** Cracking stages.

Stage (n)	Load	Cracking Pattern	Load Range
(1)	(2)	(3)	(4)
0	P	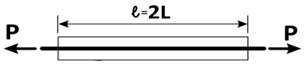	0 ≤ P < P_cr1_
1	Pcr1	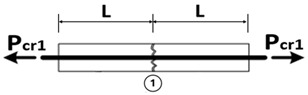	P_cr1_ ≤ P < P_cr2_
2	Pcr2	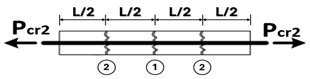	P_cr2_ ≤ P < P_cr3_
3	Pcr3	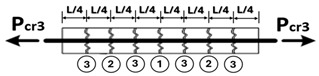	P_cr3_ ≤ P

**Table 2:** Number of cases with NCs.

		Number of Cases (Out of 10)				
	NC	3 Cracks	4 Cracks	5 Cracks	6 Cracks	7 Cracks
CoV						
**750 mm long member**						
5%			1	7	2	
10%				5	5	
20%				1	2	7

**Table 3:** Cracking loads for CoV = 5%.

		Cracking Load (kN)					
	Crack No.	1	2	3	4	5	6
Case No.							
1		19.77	19.97	22.43	28.78	45.63	
2		18.74	21.94	22.61	32.76	40.91	41.18
3		20.01	20.31	25.10	25.10	33.29	
4		19.70	20.20	24.59	27.43	39.60	
5		18.54	21.55	23.20	25.76	48.30	51.04
6		19.00	20.04	22.49	31.00	33.34	
7		19.41	20.17	22.68	26.80	47.89	
8		19.59	21.34	22.37	26.18		
9		19.58	20.61	23.62	27.01	41.46	
10		20.10	20.40	24.97	26.11	34.37	
min		18.54	19.97	22.37	25.10	33.29	41.18
max		20.10	21.94	25.10	32.76	48.30	51.04
Average		19.44	20.65	23.41	27.69	40.53	46.11
Det		**22.08**	**23.7**	**23.7**			
Average/Det		0.880	0.871	0.988			

**Table 4:** Cracking loads for CoV = 10%.

		Cracking Load (kN)					
	Crack No.	1	2	3	4	5	6
**Case No.**							
1		16.83	19.31	20.09	26.34	32.85	
2		17.63	18.75	22.24	23.42	37.91	
3		15.83	18.57	19.94	26.90	34.21	36.85
4		16.14	18.10	21.13	25.32	28.13	
5		17.04	18.84	20.66	23.49	23.63	
6		15.42	16.65	18.58	29.01	31.72	34.67
7		17.46	18.54	19.27	26.80	27.18	51.74
8		16.24	19.86	22.28	24.78	28.17	
9		17.27	18.29	22.21	25.26	32.65	35.00
10		16.43	18.51	20.80	23.79	36.23	38.39
min		15.42	16.65	18.58	23.42	23.63	34.67
max		17.63	19.86	22.28	29.01	37.91	51.74
Average		16.63	18.54	20.72	25.51	31.27	39.33
Det		**22.08**	**23.70**	**23.70**			
Average/Det		0.753	0.782	0.874			

**Table 5:** Cracking loads for CoV = 20%.

		Cracking Load (kN)						
	Crack No.	1	2	3	4	5	6	7
Case No.								
1		13.66	14.30	14.40	21.99	22.16	25.26	25.91
2		8.52	13.64	14.54	17.88	31.11	49.29	
3		11.76	13.06	13.18	18.55	20.70	32.39	36.37
4		11.41	14.03	15.53	2.165	24.73	27.63	42.15
5		13.34	14.41	17.31	20.21	21.22	23.92	26.66
6		9.80	11.82	13.97	15.19	22.65		
7		11.24	13.00	14.89	17.24	23.16	47.06	
8		12.31	14.29	14.52	16.00	19.06	40.11	40.13
9		12.13	14.34	15.02	16.99	25.19	37.48	42.26
10		12.64	13.43	14.85	20.61	21.12	26.74	40.41
min		8.52	11.82	13.18	15.19	19.06	23.92	25.91
max		13.66	14.41	17.31	21.99	31.11	49.29	42.26
Average		11.68	13.63	14.82	18.63	23.11	34.43	36.27
Det		**22.08**	**23.70**	**23.70**				
Average/Det		0.529	0.575	0.625				

## Data Availability

The original contributions presented in the study are included in the article. Further inquiries can be directed to the corresponding author.
